# A unique sessile loricate euglenid, *Lepocinclis loricata* sp. nov. (Euglenophyta, Phacaceae), from South Africa: Evolutionary implications

**DOI:** 10.1111/jpy.70163

**Published:** 2026-04-15

**Authors:** Bożena Zakryś, Sanet Janse van Vuuren, Aleksandra Kluczek, Maja Łukomska‐Kowalczyk

**Affiliations:** ^1^ Faculty of Biology, Biological and Chemical Research Centre University of Warsaw Warsaw Poland; ^2^ Unit for Environmental Sciences and Management North‐West University Potchefstroom South Africa

**Keywords:** environmental sampling, Euglenida, *Lepocinclis loricata* sp. nov., morphology, phylogeny

## Abstract

In this paper, we describe *Lepocinclis loricata* sp. nov. (Phacaceae), a sessile loricate euglenid that differs from all known loricate taxa by its unique combination of the presence of a lorica, monad morphology, sessile habit, and phylogenetic position within the Phacaceae. Populations of this species inhabit two turbid, silt‐rich waterbodies, namely an artificial impoundment in the Western Cape Province and a natural, small river in the Free State Province, South Africa. The cylindrical, transparent loricae of *L. loricata* sp. nov. are attached to fine clay and silt particles suspended in the water column. Each lorica encloses a rigid, spindle‐shaped monad containing numerous chloroplasts without pyrenoids and two large, rod‐like paramylon grains. Phylogenetic analyses place *L. loricata* sp. nov. within the *Lepocinclis* clade, representing a loricate euglenid identified in the family Phacaceae. This discovery provides new insight into the diversification and adaptive evolution of photosynthetic euglenids and the independent emergence of sessility within the group.

**Abbreviations**
GTR: general time reversible
LM: light microscopy
ML: maximum likelihood
PCR: polymerase chain reaction
rbs: rapid bootstrap
SEM: scanning electron microscopy

## INTRODUCTION

Euglenids are a diverse group within the Euglenozoa, classified in the Discoba lineage (Burki et al., 2020). They inhabit a wide range of aquatic environments, from marine to freshwater, and exhibit an exceptional diversity of nutritional modes, including phagotrophy, osmotrophy, mixotrophy, and photoautotrophy (Kostygov et al., 2021; Yamaguchi et al., 2012). Most euglenids are unicellular, free‐living flagellates with cells surrounded only by a pellicle, which comprises the plasma membrane, protein strips, subtending microtubules, and endoplasmic reticulum (Esson & Leander, 2006).

Among photosynthetic euglenids, five genera are particularly distinctive in terms of morphology and life history: *Parastrombomonas*, *Strombomonas*, and *Trachelomonas*, which have monads enclosed within loricae (Fells et al., 2023); *Colacium*, which exhibits a biphasic life cycle alternating between sessile and motile stages (Ehrenberg, 1835; Rosowski & Kugrens, 1973); and *Ascoglena*, with monads that are both sessile and loricate (Stein, 1878).

The production of a lorica, a rigid extracellular envelope surrounding the monad, is one of the key morphological innovations within some photosynthetic euglenids. Loricate taxa, traditionally classified in the Euglenaceae, include *Parastrombomonas*, *Strombomonas*, and *Trachelomonas*, all of which possess free‐swimming monads enclosed within mineralized loricae (Fells et al., 2023). These structures are believed to provide protection and mechanical stability, enabling survival under fluctuating environmental conditions. Sessile loricate forms, however, are exceedingly rare. To date, only *Ascoglena* and *Trachelomonas sessilis* have been described as sessile loricate euglenids, both known solely from historical reports that have never been verified using modern techniques. Consequently, our understanding of the origin, evolution, and adaptive significance of sessility and lorica formation within the Euglenophyta remains limited.

Whereas *Colacium*, *Parastrombomonas*, *Strombomonas*, and *Trachelomonas* are species‐rich (particularly *Trachelomonas*), widespread, and phylogenetically well resolved, little is known about the genus *Ascoglena*. It remains poorly studied and includes only four recognized species; its phylogenetic position is still unresolved due to the absence of molecular data. The monads of *Ascoglena* are morphologically similar to those of *Euglena*: They are highly metabolic (capable of changing shape), contain only small paramylon grains, and possess chloroplasts with pyrenoids—either a single, star‐shaped chloroplast in *A. viridis* or numerous chloroplasts in *A. amphoroides*, *A. vaginicola*, and *A. kumaraii*. The loricae surrounding the monads are thin‐walled and broad (ovoid to fusiform), and each possesses a large apical opening (Stein, 1878). Based on similar lorica morphology, *T. sessilis* described from Australia (Playfair, 1915) may also belong to *Ascoglena*. Unfortunately, the monad morphology was not described in the protologue, and the species has not been rediscovered since its original description.

Recently, a sessile loricate euglenid was observed that differed from all previously known loricate taxa based on its unique combination of lorica structure, cell morphology, sessile habit, and phylogenetic placement within the Phacaceae. In this manuscript, we describe this new species using an integrative approach combining morphological observations and molecular data. We provide a detailed account of its morphology, ecology, and phylogenetic position within the Phacaceae. These findings contribute new insight into the diversification and adaptive evolution of photosynthetic Euglenids, including the independent emergence of sessility within the group.

## MATERIALS AND METHODS

### Study area

**FIGURE 1:**
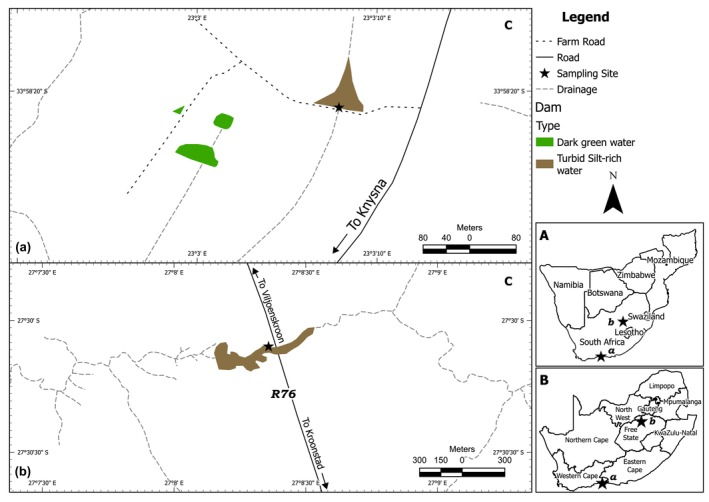
Location of the sampling sites near Knysna (Western Cape Province, South Africa) and Kroonstad (Free State Province, South Africa). (A) Map of southern Africa showing the position of the sampling sites. (B) Map of South Africa indicating the provinces where the sampling sites are located. (C) Enlarged view of the areas surrounding the two sampling sites—(a) site near Knysna and (b) site near Kroonstad.

Sampling was undertaken at two sampling sites in South Africa (Figure 1). The first site is an artificial impoundment (coordinates: 33°58′20.7″ S 23°03′07.7″ E; Figure 1a), located on the remainder of Farm 99 Gouna, ~6.9 km north of Knysna and ~607 m west of the Gouna River in the Western Cape Province, South Africa (Figure 1c, map a). The impoundment lies in the upper reaches of a non‐perennial stream draining southeast toward the Gouna River.

Initially, only a small pond was present at this location, but during 2021, after evacuation and clearance of all vegetation (especially *Eucalyptus* trees) in the surrounding area, the waterbody was enlarged during the upgrading of an access road crossing a non‐perennial stream. The enlargement of the waterbody was intended to provide additional water for irrigation, but it was never used for this purpose due to regulatory constraints. The basin, composed entirely of unlined natural clay, was excavated to a depth of ~3 m but currently holds water to a depth of ~1–2 m. Its estimated surface area is 2225 m^2^, with an approximate volume of 1780 m^3^. Since its modification, the impoundment has generally retained water throughout the year, although fluctuations occur depending on rainfall and ongoing rehabilitation activities. The water is turbid and has a characteristic light brown color caused by suspended silt and clay particles. No submerged or emergent macrophytes were recorded in the pond at the time of sampling. The altitude of the sampling site is 237 m above mean sea level. Rainfall occurs year round with seasonal peaks in spring and autumn.

A second water sample was collected from a bridge on the R76 road (coordinates: 27°30′06.6″ S, 27°08′21.8″ E) crossing the Doringspruit River (Figure 1c, map b). The sampling site is situated between the towns of Viljoenskroon and Kroonstad in the Free State Province, South Africa (~39.4 km southeast of Viljoenskroon and 19.3 km northwest of Kroonstad) and is located near the inflow of the Doringspruit River into a small, downstream impoundment. The surrounding landscape is gently undulating and predominantly agricultural, consisting of cultivated croplands and grazing pastures, with a narrow band of riparian vegetation along the stream. The water at the sampling site was visibly turbid and rich in fine, suspended silt particles, likely influenced by upstream soil disturbance and agricultural runoff. Although diffuse nutrient inputs from surrounding land use may influence the chemical characteristics of the stream, the site still receives full sunlight exposure, providing suitable conditions for algal growth.

The presence of fine particles and the silty nature of both sampling sites form substrates on which algae can settle and proliferate.

### Sample collection and morphological study

Plankton samples were collected in March and April 2025, then filtered using a plankton net with a mesh size of 10 μm to concentrate the algae. The physicochemical water parameters were measured using a Hanna Instruments HI98129 multiparameter. The samples were transported to the laboratories of the North‐West University in Potchefstroom (South Africa) and kept in the laboratory in a 750‐mL glass jar for ~2 weeks. During this time, cells of the new species were isolated using a micromanipulator (MM‐89 Narishige) with an attached micropipette, mounted on a Nikon Eclipse E‐600 microscope (Nikon, Tokyo, Japan). The isolated cells were then transferred through several drops of sterile media for sample purification, followed by photographing and cell measuring. Thereafter, the samples were frozen at −80°C for DNA isolation.

Measurements were done with light microscopy (LM) photographs, and video clips of the isolated cells were taken with a NIKON Eclipse E‐600 microscope, equipped with the software NIS Elements BR v.3.1 (Nikon) and a NIKON DX‐1200 digital camera for image recording and processing. Lorica and monad length and width were measured for 50 individuals.

Given that lorica structure and surface ornamentation are key features for identification, samples were also prepared for scanning electron microscopy (SEM). A droplet of the sample was placed on circular glass coverslips (12‐mm diameter) and allowed to air‐dry for 7 days. The coverslips were then transferred to a desiccator for at least 24 h. Subsequently, they were mounted on aluminum SEM stubs using carbon tape and sputter‐coated with gold–palladium for 90 s using an SPI Module Sputter Coater (SPI Supplies, West Chester, Pennsylvania, United States). The SEM observations were performed with a Phenom Pro Desktop SEM (Phenom‐World BV, Eindhoven, Netherlands).

### DNA isolation, amplification, and sequencing

DNA extraction was performed using the Chelex 100 (Bio‐Rad, Hercules, California, United States) chelating resin according to the protocol in Fells et al. (2023). For polymerase chain reaction (PCR) amplification and purification of three genes (nSSU rDNA, cpLSU rDNA, and cpSSU rDNA), the previously described protocol (Łukomska‐Kowalczyk et al., 2021; Zakryś et al., 2002) and primers (Zakryś et al., 2024) were used.

Most of the PCR products were Sanger sequenced as described previously (Zakryś et al., 2002). The cpLSU gene of the new species was purified and barcoded using the ligation kit SQK‐NBD114.24 (Oxford Nanopore Technologies, Oxford, Great Britain) according to the manufacturer's instructions and sequenced using a MinION device (Oxford Nanopore Technologies). Raw Nanopore sequencing data were base‐called and demultiplexed using MinKNOW software (analysis mode), filtered using filtlong (v 0.2.0; Wick, 2022), clustered by vsearch (Rognes et al., 2016), and polished using racon (Vaser et al., 2017) and minmap2 (Li, 2018).

### Sequence accession numbers, alignments, and sequence analyses

Fifteen new sequences were submitted to GenBank under the accession numbers PX714901–PX714915 (Table S1). These included nSSU, cpSSU, and cpLSU rDNA gene sequences. In addition, seven cpSSU and five cpLSU rDNA gene sequences from other *Lepocinclis* and *Phacus* species were obtained to ensure broad representation of these genera, which were identified as the closest relatives of the new species based on preliminary analyses.

The sequences of each gene (104 nSSU, 86 cpLSU, 88 cpSSU rDNA) were aligned separately using Fast Statistical Alignment (v. 1.15.9) with default parameters (Bradley et al., 2009). Regions of doubtful homology were trimmed with TrimAl v.1.2 with the option, automated1 (Capella‐Gutierrez et al., 2009). After removal, 1595, 1499, and 1094 nt were left for the nSSU, cpLSU, and cpSSU rDNA genes, respectively. The final data set was concatenated using SeaView v.5 (Gouy et al., 2010) and equaled 4188 nt.

The combined data set generated for the analyses consisted of 105 sequences, representing 74 euglenid species; of these, three sequences of Eutreptiales were used as an outgroup to root the tree. The maximum likelihood (ML) analyses were carried out using RAxML v.8 (Stamatakis, 2014) with the relevant model applied to each partition and support evaluated using bootstrapping (1000 replicates) and allowing for each partition to have individual α‐shape parameters, general time reversible (GTR) rates, and empirical base frequencies parameters estimated.

## RESULTS

**FIGURE 2:**
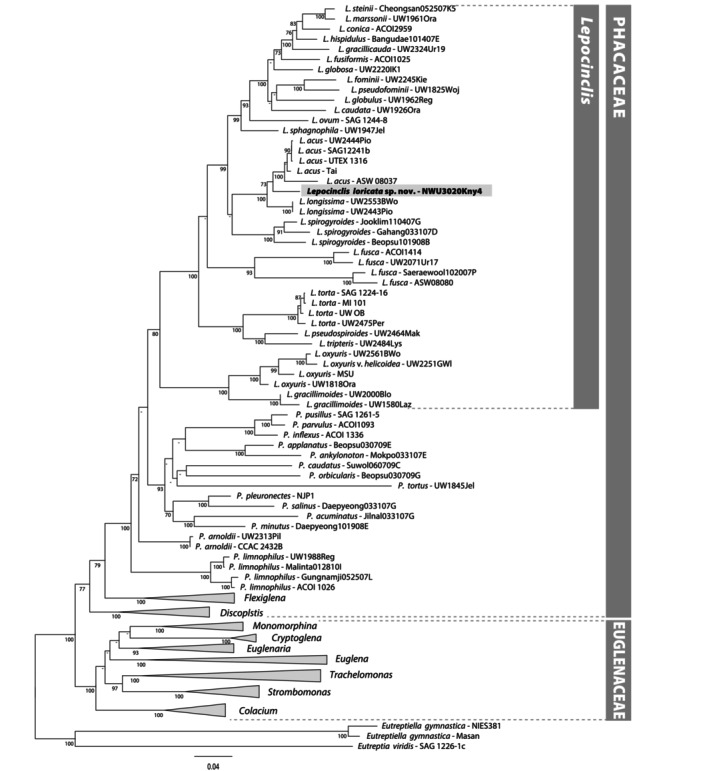
Maximum‐likelihood phylogenetic tree based on 104 of nSSU rDNA, 88 of cpSSU rDNA, and 86 of cpLSU rDNA genes representing 105 strains or isolates. Clades containing all genera except *Lepocinclis* and *Phacus* are collapsed. Nodes are labeled with rapid bootstrap (rbs) values; values below 70 are indicated by a hyphen (−). The scale bar represents the number of substitutions per site.

In the phylogenetic tree, the new species formed a sister lineage to the maximally supported *Lepocinclis acus* clade, and together these taxa constituted a well‐supported clade with *L. longissima* (Figure 2; Figure S1). Morphologically, the new taxon differs from *L. acus* and *L. longissima* not only by the presence of a lorica, but also by monad traits, including much smaller size (~30–50 μm in length) and the presence of only two large paramylon grains, whereas the latter two species possess much larger cells (100–300 μm in length) and several large paramylon grains.

All other lorica forming species are grouped in the *Trachelomonas* and *Strombomonas* clade, both in the Euglenaceae family.

***Taxonomy***

**FIGURE 3:**
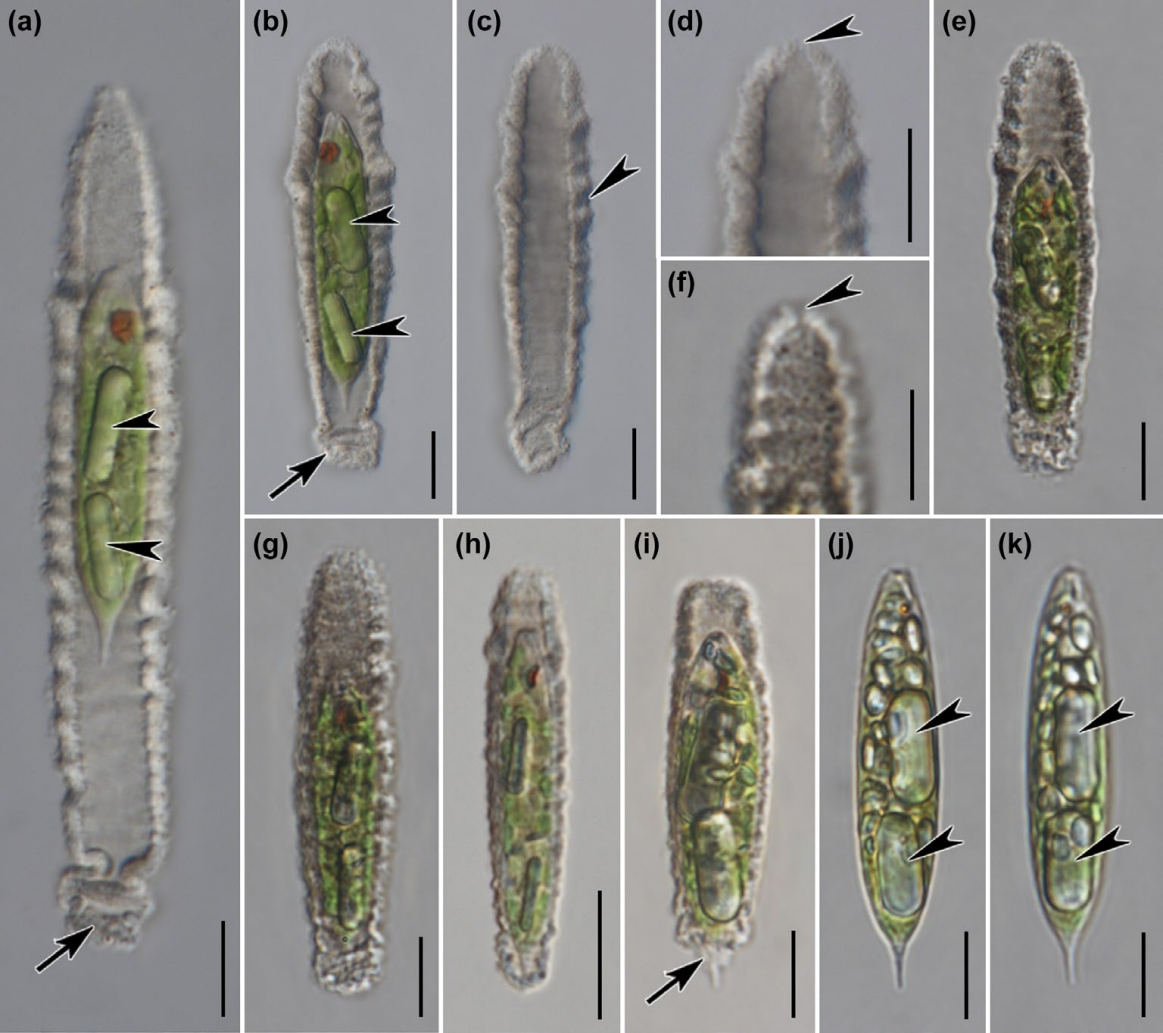
LM microphotographs showing an overview of living cells of *Lepocinclis loricata* sp. nov. (a, b) Cigar‐shaped lorica attached to a particle (arrow) with a monad inside; the monad contains two large, rod‐like paramylon grains (arrowheads). (c) Empty lorica with transverse folds of its wall visible (arrowhead). (d, f) Anterior end of the lorica with a small aperture through which a single flagellum emerges (arrowheads). (e, g, h) Individuals with intact loricae, with monads visible inside. (i) Individual with a damaged posterior end of the lorica; the monad protruding from the broken lorica is visible (arrow). (j, k) Monad lacking a lorica, with two large, rod‐like paramylon grains visible inside (arrowheads). Scale bars = 10 μm.

**FIGURE 4:**
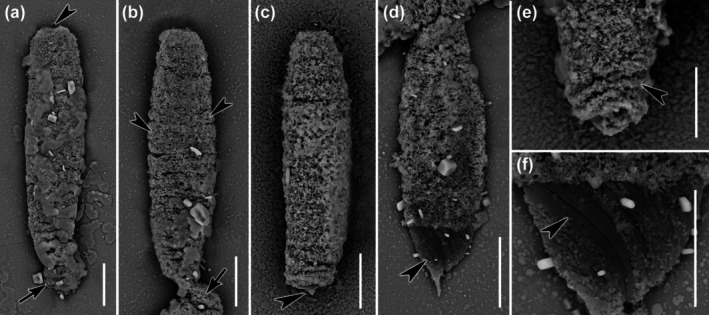
SEM micrographs showing the lorica of *Lepocinclis loricata* sp. nov.; the lorica surface is slightly textured and occasionally covered with adhering clay or silt particles. (a, b) Lorica attached to a particle (arrow); in (a) a small aperture is visible at the top of the anterior end (arrowhead); in (b) transverse folds of the wall are visible (arrowheads). (c) Detached lorica with a slightly damaged posterior end (arrowhead). (d) Severely damaged lorica with a monad protruding through a wide rupture (arrowhead). (e) Fragment of the posterior end showing folds of the lorica wall (arrowhead). (f) Fragment of the monad protruding from a damaged lorica; wide periplast bands of the monad are clearly visible (arrowhead). Scale bars = 10 μm.

***Lepocinclis loricata*** sp. nov. Zakryś, Janse van Vuuren and Łukomska (Figures 3, 4).

**Description**: Cells (monads) spindle‐shaped and rigid (27–55 μm long and 6.5–14.5 μm wide), enclosed within rigid loricae. Numerous discoid chloroplasts are present, lacking pyrenoids. Paramylon grains dimorphic: two large, rod‐like paramylon grains accompanied by numerous smaller granules scattered throughout the cytoplasm (Figure 3a,b,j,k). Loricae cigar‐shaped (narrow‐cylindrical), transparent, 43–83 μm long, and 9.6–16.4 μm wide, with regularly arranged transverse folds. The lorica surface is slightly textured and occasionally covered with adhering clay or silt particles. The anterior end bears a small aperture through which a single flagellum emerges (Figures 3d and 4a). The posterior end of the lorica is attached to fine clay or silt particles suspended in the water column. Monads are nonmotile within the lorica, but are capable of swimming when released from the lorica cavity. Loricae occur singly or, less frequently, in small clusters attached to common suspended particles. Figure 3b represents an illustration of the type. Representative DNA sequences: GenBank cpSSU rDNA: PX714914; nSSUrDNA: PX714915; cpLSU rDNA: PX714901.

Holotype: SANDC 25–123 (North‐West University [Potchefstroom Campus], South Africa) ~100 individuals isolated from an environmental sample, permanently preserved in 50% ethanol, in the South African National Diatom Collection. Artificial impoundment, Western Cape Province, South Africa (33°58'20.7" S, 23°03'07.7" E); April 10, 2025, *leg*. *B.Zakryś*, *S.Janse van* *Vuuren*, *M.Łukomska‐Kowalczyk*.

**Etymology**: The specific epithet *loricata* refers to the presence of a lorica, which represents the most distinctive morphological feature of the new species.

**Habitat:** Planktonic or benthic; cells attached to suspended fine clay and silt particles. Water parameters: pH = 6.57, EC (electrical conductivity) = 214 μS · cm^−1^ and temperature = 31.2°C.

## DISCUSSION

The discovery of *Lepocinclis loricata* sp. nov. in South Africa highlights that the morphological and evolutionary diversity of euglenids on the African continent is substantially underestimated. Although photosynthetic euglenids are globally diverse, research efforts in Africa, particularly in southern Africa, have historically been limited, largely due to the small number of specialists working in the region. Existing studies are sporadic and geographically uneven, with published records concentrated in a few countries, and vast areas remain poorly surveyed (Janse van Vuuren et al., 2025). A recent checklist of freshwater photosynthetic euglenids from southern Africa reported only 154 taxa, in contrast to more than 2000 species currently recognized worldwide (Guiry & Guiry, 2025; Levanets et al., 2025). Within this assemblage, *Lepocinclis* is currently represented by only 17 species regionally, compared with 91 species accepted globally.

This pronounced disparity between recorded and global diversity strongly indicates that many African euglenid taxa remain undescribed or undetected. The occurrence of a morphologically and evolutionarily unusual taxon such as *Lepocinclis loricata* sp. nov., representing an independent emergence of sessility within the Phacaceae, supports the view that African freshwater systems may harbor disproportionately higher numbers of novel or rare evolutionary forms than currently recognized. Targeted taxonomic, molecular, and ecological studies across underexplored regions of the continent are therefore essential to better resolve patterns of diversification, character evolution, and biogeography within the Euglenophyta.

Sessile loricate euglenids represent one of the rarest and least understood morphotypes among photosynthetic Euglenophyta (Kostygov et al., 2021). To date, only two taxa, namely *Ascoglena* (four species) and *Trachelomonas sessilis*, have been described as sessile and loricate. *Lepocinclis loricata* sp. nov. differs markedly in morphology from both these taxa. Its loricae are transparent, narrowly cylindrical and transversely folded, and each lorica bears a small apical aperture. The monads are rigid and spindle‐shaped, and they contain two large rod‐shaped paramylon grains together with numerous discoid chloroplasts lacking pyrenoids, features characteristic of the Phacaceae. These traits, particularly the presence of two large rod‐like paramylon grains, recall those of *Phacus limnophilus* (Łukomska‐Kowalczyk et al., 2020). It was, therefore, initially expected that *Lepocinclis loricata* sp. nov. would be closely related to *P. limnophilus* and that they would potentially form a distinct phylogenetic lineage. The current placement of *P. limnophilus* within the *Phacus* clade was not strongly supported across all phylogenetic analyses. In some trees, the species appeared as the earliest‐branching lineage of the genus, occasionally together *with P. arnoldii*, although the clade of the genus was not maximally supported in all analyses (Karnkowska et al., 2015; Kim et al., 2015; Łukomska‐Kowalczyk et al., 2020, 2021). In other phylogenetic trees, *P. limnophilus* fell outside the clade, rendering the genus paraphyletic due to the inclusion of *Lepocinclis* representatives (Kim & Shin, 2014). Moreover, morphologically *P. limnophilus* more closely resembles members of *Lepocinclis* than *Phacus*, as evidenced by its non‐flattened, fusiform cells and large, rod‐shaped paramylon grains (Kim et al., 2015; Łukomska‐Kowalczyk et al., 2020, 2021). In the analysis presented here, *P. limnophilus* was placed outside the *Phacus* clade, as was *P. arnoldii*, suggesting that phylogenetic relationships within the family Phacaceae remain unresolved and urgently require further study.

The second hypothesis was that *Lepocinclis loricata* sp. nov. would cluster within the *Strombomonas* clade. This expectation was based on the existence of *S. taiwanensis* (Yamagishi & Couté, 1995), a species with similarly unusual morphology, having a transparent, narrowly fusiform lorica that lacks a distinct neck, wherein the monad possesses two large paramylon grains; unfortunately, no information on its chloroplasts is available. However, phylogenetic analyses placed *Lepocinclis loricata* sp. nov. firmly within the *Lepocinclis* clade, indicating that lorica formation and sessility evolved independently within phototrophic euglenids. This finding provides new insights into the diversification and adaptive evolution of the Euglenophyta.

The monads of *Ascoglena* exhibit a typical *Euglena*‐like structure: They are highly metabolic, possess only small paramylon grains, and contain one to several chloroplasts with pyrenoids (Stein, 1878). Although the phylogenetic position of *Ascoglena* remains unresolved, morphological characteristics suggest that *Ascoglena* likely belongs to the Euglenaceae.

As in other loricate euglenids (*Strombomonas, Trachelomonas*), cell division in *Lepocinclis loricata* sp. nov. likely occurs within the lorica, with the daughter cell exiting through the apical opening, although this process was not observed. Occasionally, loricae are twice as long as the enclosed monad (Figure 2a), which may indicate gradual elongation as a consequence of successive cell divisions. When a lorica becomes mechanically detached from the substrate, the monad escapes through a posterior rupture and resumes actively swimming, a behavior repeatedly observed under laboratory conditions during the current study (Figures 2i and 3d,f).

*Lepocinclis loricata* sp. nov. appears to be rare in South Africa, having been recorded in only two turbid waterbodies despite extensive surveys of more than 150 comparable sites across the Eastern Cape, Free State, Gauteng, North West, Western Cape, and Limpopo provinces conducted during March and April 2025. The presence of a sessile, loricate representative within the Phacaceae indicates that lorica formation and sedentary life strategies evolved multiple times and under different selective pressures within phototrophic euglenids. The unique combination of sessility and the presence of a lorica may be associated with the occurrence in shallow, particularly turbid ponds. This lifestyle likely provides protection against mechanical damage: Sessility reduces the frequency of collisions with suspended particles, and the lorica mitigates the impact of such collisions. An additional advantage of a sessile lifestyle in turbid waters with low light penetration may be the ability to remain attached near the surface (e.g., to plant stems or rocks), thereby avoiding sinking into darker layers. Furthermore, the lorica may function as a light filter, protecting cells from excessive UV radiation during brief exposure to intense sunlight, which may be especially important in hot climates or during periods of fluctuating water levels. Strong predation pressure may represent another selective factor favoring both lorica formation and sessility. Further molecular and ultrastructural studies are required to clarify the evolutionary mechanisms underlying lorica development and to reassess phylogenetic relationships among loricate and non‐loricate taxa within the group.

## AUTHOR CONTRIBUTIONS

**Bożena Zakryś:** Conceptualization (equal); data curation (equal); funding acquisition (lead); investigation (equal); supervision (equal); visualization (equal); writing – original draft (lead); writing – review and editing (equal). **Sanet Janse van Vuuren:** Data curation (equal); funding acquisition (supporting); investigation (equal); resources (equal); visualization (equal); writing – original draft (supporting); writing – review and editing (equal). **Aleksandra Kluczek:** Investigation (equal). **Maja Łukomska‐Kowalczyk:** Conceptualization (equal); data curation (equal); formal analysis (lead); investigation (equal); methodology (equal); supervision (equal); visualization (equal); writing – original draft (supporting); writing – review and editing (equal).

## Supporting information


**Figure S1.** Maximum‐likelihood phylogenetic tree based on 104 of nSSU rDNA, 88 of cpSSU rDNA, and 86 of cpLSU rDNA genes representing 105 strains or isolates. Nodes are labeled with the rapid bootstrap (rbs) values. Scale bar represents number of substitutions per site.

**Table S1.** List of species and sampling data of isolates/strains used in this study. GenBank accession numbers and their nSSU, nLSU, and cpLSU rDNA genes sequences are given, with new sequences indicated in bold type.

## References

[jpy70163-bib-0001] Bradley, R. K. , Roberts, A. , Smoot, M. , Juvekar, S. , Do, J. , Dewey, C. , Holmes, I. , & Pachter, L. (2009). Fast statistical alignment. PLoS Computational Biology, 5(5), e1000392. 10.1371/journal.pcbi.1000392 19478997 PMC2684580

[jpy70163-bib-0002] Burki, F. , Roger, A. J. , Brown, M. W. , & Simpson, A. G. B. (2020). The new tree of eukaryotes. Trends in Ecology & Evolution, 35, 43–55. 10.1016/j.tree.2019.08.008 31606140

[jpy70163-bib-0003] Capella‐Gutierrez, S. , Silla‐Martinez, J. M. , & Gabaldon, T. (2009). TrimAl: A tool for automated alignment trimming in large scale phylogenetic analyses. Bioinformatics, 25, 1972–1973. 10.1093/bioinformatics/btp348 19505945 PMC2712344

[jpy70163-bib-0004] Ehrenberg, C. G. (1835). Dritter Beitrag zur Erkenntniss grosser Organisation in der Richtung des kleinsten Raumes. Abhandlungen der Königlichen Akademie der Wissenschaften zu Berlin, 1833, 145–336.

[jpy70163-bib-0005] Esson, H. J. , & Leander, B. S. (2006). A model for the morphogenesis of strip reduction patterns in phototrophic euglenids: Evidence for heterochrony in pellicle evolution. Evolution & Development, 8, 378–388. 10.1111/j.1525-142X.2006.00110.x 16805902

[jpy70163-bib-0006] Fells, A. , Jiang, X. , Jankowska, K. , Łukomska‐Kowalczyk, M. , Milanowski, R. , Wang, Q. , & Zakryś, B. (2023). Molecular and morphological delimitation of species in *Strombomonas* (Euglenaceae) including a protocol for DNA isolation utilising a chelating resin. Taxon, 72, 733–750. 10.1002/tax.12937

[jpy70163-bib-0008] Gouy, M. , Guindon, S. , & Gascuel, O. (2010). SeaView version 4: A multiplatform graphical user interface for sequence alignment and phylogenetic tree building. Molecular Biology and Evolution, 27, 221–224. 19854763 10.1093/molbev/msp259

[jpy70163-bib-0009] Guiry, M. D. , & Guiry, G. M. (2025). AlgaeBase. World‐wide electronic publication. National University of Ireland, Galway. http://www.algaebase.org

[jpy70163-bib-0010] Janse Van Vuuren, S. , Łukomska‐Kowalczyk, M. , & Zakryś, B. (2025). Filling gaps in the taxonomy of loricate euglenids (Euglenophyta): Scanning electron microscopy studies of *Strombomonas* and *Trachelomonas* from Angola, including the description of *T. angolensis* sp. nov. South African Journal of Botany, 185, 769–797. 10.1016/j.sajb.2025.08.013

[jpy70163-bib-0011] Karnkowska, A. , Bennett, M. S. , Watza, D. , Kim, J. I. , Zakryś, B. , & Triemer, R. E. (2015). Phylogenetic relationships and morphological character evolution of photosynthetic Euglenids (Excavata) inferred from taxon‐rich analyses of five genes. The Journal of Eukaryotic Microbiology, 62, 362–373. 25377266 10.1111/jeu.12192

[jpy70163-bib-0012] Kim, J. I. , Linton, E. W. , & Shin, W. (2015). Taxon‐rich multigene phylogeny of the photosynthetic euglenoids (Euglenophyceae). Frontiers in Ecology and Evolution, 3, 98. 10.3389/fevo.2015.00098

[jpy70163-bib-0013] Kim, J. I. , & Shin, W. (2014). Molecular phylogeny and cryptic diversity of the genus *Phacus* (Phacaceae, Euglenophyceae) and the descriptions of seven new species. Journal of Phycology, 50, 948–959. 26988648 10.1111/jpy.12227

[jpy70163-bib-0014] Kostygov, A. Y. , Karnkowska, A. , Votýpka, J. , Tashyreva, D. , Maciszewski, K. , Yurchenko, V. , & Lukeš, J. (2021). Euglenozoa: Taxonomy, diversity and ecology, symbioses and viruses. Open Biology, 11, 200407. 10.1098/rsob.200407 33715388 PMC8061765

[jpy70163-bib-0015] Levanets, A. , Janse van Vuuren, S. , Łukomska‐Kowalczyk, M. , & Zakryś, B. (2025). Annotated checklist of photosynthetic freshwater euglenids (Euglenales: Euglenaceae and Phacaceae) from southern Africa. Phytotaxa, 710, 22–56. 10.11646/phytotaxa.710.1.2

[jpy70163-bib-0016] Li, H. (2018). Minimap2: Pairwise alignment for nucleotide sequences. Bioinformatics, 34, 3094–3100. 10.1093/bioinformatics/bty191 29750242 PMC6137996

[jpy70163-bib-0017] Łukomska‐Kowalczyk, M. , Chaber, K. , Fells, A. , Milanowski, R. , & Zakryś, B. (2021). Description of *Flexiglena* gen. nov. and new members of *Discoplastis* and *Euglenaformis* (Euglenida). Journal of Phycology, 57, 766–779. 10.1111/jpy.13107 33205421 PMC8248102

[jpy70163-bib-0018] Łukomska‐Kowalczyk, M. , Fells, A. , Chaber, K. , Milanowski, R. , & Zakryś, B. (2020). Taxon‐rich phylogeny and taxonomy of the genus *Phacus* (Euglenida) based on morphological and molecular data. Journal of Phycology, 56, 1135–1156. 10.1111/jpy.13028 32428982 PMC7687149

[jpy70163-bib-0029] Playfair, G. I. (1915). The genus Trachelomonas. Proceedings of the Linnean Society of New South Wales, 40, 1–41.

[jpy70163-bib-0019] Rognes, T. , Flouri, T. , Nichols, B. , Quince, C. , & Mahé, F. (2016). VSEARCH: A versatile open source tool for metagenomics. PeerJ, 4, e2584. 10.7717/peerj.2584 27781170 PMC5075697

[jpy70163-bib-0020] Rosowski, J. R. , & Kugrens, P. (1973). Observations on the euglenoid *Colacium* with special reference to the formation and morphology of attachment material. Journal of Phycology, 9, 370–383. 10.1111/j.1529-8817.1973.tb04110.x

[jpy70163-bib-0021] Stamatakis, A. (2014). *R*AxML version 8: A tool for phylogenetic analysis and post‐analysis of large phylogenies. Bioinformatics, 30, 1312–1313. 10.1093/bioinformatics/btu033 24451623 PMC3998144

[jpy70163-bib-0022] Stein, F. (1878). Der Organismus der Infusionsthiere nach eigenen Forschungen in systematischere Reihenfolge bearbeitet. III. Abtheilung. Die Naturgeschichte der Flagellaten oder Geisselinfusorien. I. Hälfte. W. Engelmann.

[jpy70163-bib-0023] Vaser, R. , Sović, I. , Nagarajan, N. , & Šikić, M. (2017). Fast and accurate de novo genome assembly from long uncorrected reads. Genome Research, 27, 737–746. 10.1101/gr.214270.116 28100585 PMC5411768

[jpy70163-bib-0024] Wick, R. (2022). Filtlong. https://github.com/rrwick/Filtlong

[jpy70163-bib-0025] Yamagishi, T. , & Couté, A. (1995). *Strombomonas taiwanensis* nov. sp. (Euglenophyta, Euglenophyceae). Cryptogamie, Algologie, 16, 255–262.

[jpy70163-bib-0026] Yamaguchi, A. , Yubuki, N. , & Leander, B. S. (2012). Morphostasis in a novel eukaryote illuminates the evolutionary transition from phagotrophy to phototrophy: Description of *Rapaza viridis* n. gen. et n. sp. (Euglenozoa, Euglenida). BMC Evolutionary Biology, 12, 29. 10.1186/1471-2148-12-29 22401606 PMC3374381

[jpy70163-bib-0027] Zakryś, B. , Jankowska, K. , Majerowicz, A. , Fells, A. , & Łukomska‐Kowalczyk, M. (2024). Discovery of a new photosynthetic euglenoid in Poland: *Euglena mazurica* sp. nov. (Euglenales Euglenaceae). Protist, 175, 126015. 10.1016/j.protis.2024.126015 38301533

[jpy70163-bib-0028] Zakryś, B. , Milanowski, R. , Empel, J. , Borsuk, P. , Gromadka, R. , & Kwiatowski, J. (2002). Two different species of *Euglena, E. geniculata* and *E. myxocylindracea* (Euglenophyceae), are virtually genetically and morphologically identical. Journal of Phycology, 38, 1190–1199. 10.1046/j.1529-8817.2002.02020.x

