# Supplementary figures and images for: A unique sessile loricate euglenid, Lepocinclis loricata sp. nov. (Euglenophyta, Phacaceae), from South Africa: Evolutionary implications
Source: J Phycol. 2026 Apr 15;62(3):846–54. doi: 10.1111/jpy.70163 (PMC13280771; doi:10.1111/jpy.70163)

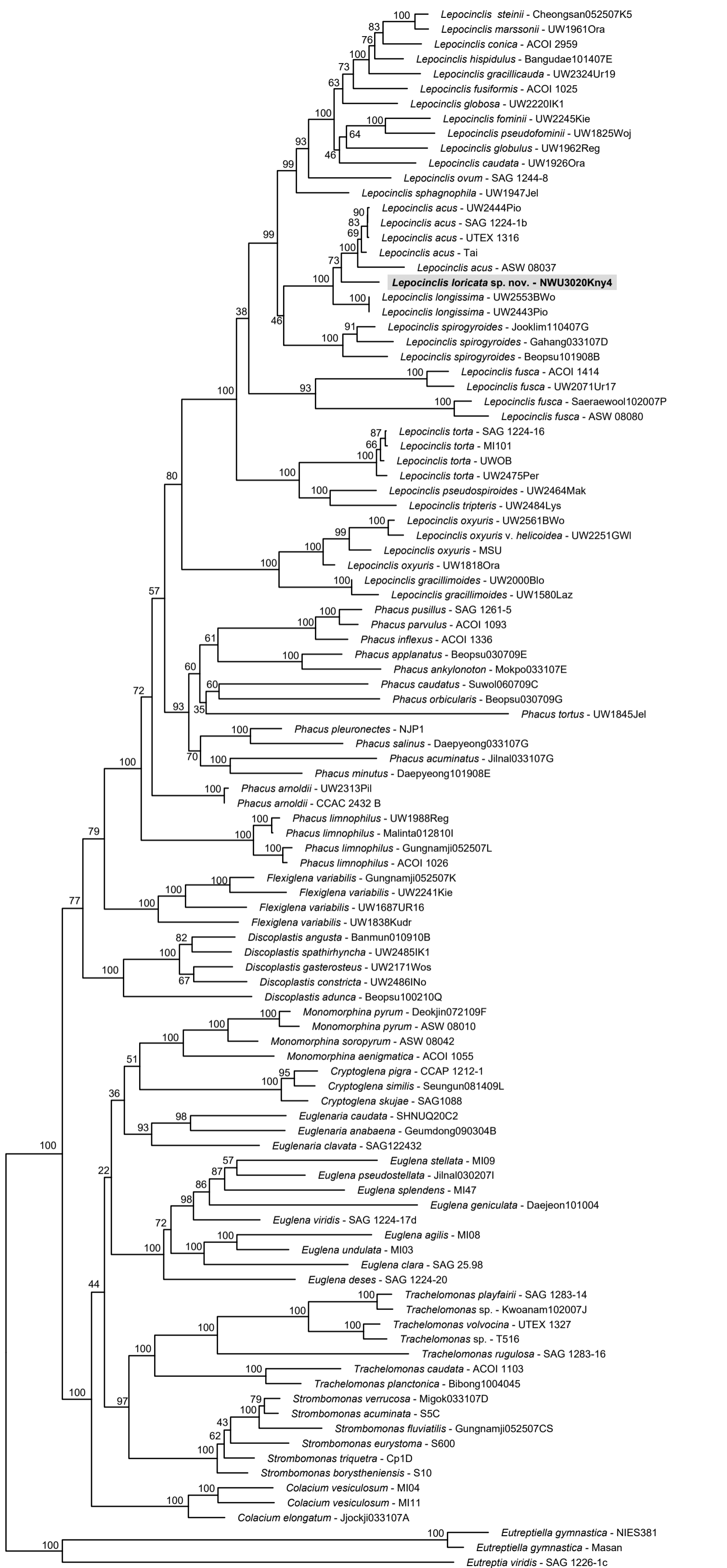

0.04

Supplement: Supplementary file 1 — Figure S1. Maximum‐likelihood phylogenetic tree based on 104 of nSSU rDNA, 88 of cpSSU rDNA, and 86 of cpLSU rDNA genes representing 105 strains or isolates. Nodes are labeled with the rapid bootstrap (rbs) values. Scale bar represents number of substitutions per site. [file JPY-62-846-s002.pdf]
